# Coordinated Modulation of Energy Metabolism and Inflammation by Branched-Chain Amino Acids and Fatty Acids

**DOI:** 10.3389/fendo.2020.00617

**Published:** 2020-09-08

**Authors:** Zhenhong Ye, Siyu Wang, Chunmei Zhang, Yue Zhao

**Affiliations:** ^1^Department of Obstetrics and Gynecology, Center for Reproductive Medicine, Peking University Third Hospital, Beijing, China; ^2^National Clinical Research Center for Obstetrics and Gynecology (Peking University Third Hospital), Beijing, China; ^3^Key Laboratory of Assisted Reproduction (Peking University), Ministry of Education, Beijing, China; ^4^Beijing Key Laboratory of Reproductive Endocrinology and Assisted Reproductive Technology, Peking University, Beijing, China; ^5^Research Units of Comprehensive Diagnosis and Treatment of Oocyte Maturation Arrest, Chinese Academy of Medical Sciences, Beijing, China

**Keywords:** branched-amino acids, fatty acids, energy metabolism, inflammation, insulin resistance, mitochondrial biogenesis

## Abstract

As important metabolic substrates, branched-chain amino acids (BCAAs) and fatty acids (FAs) participate in many significant physiological processes, such as mitochondrial biogenesis, energy metabolism, and inflammation, along with intermediate metabolites generated in their catabolism. The increased levels of BCAAs and fatty acids can lead to mitochondrial dysfunction by altering mitochondrial biogenesis and adenosine triphosphate (ATP) production and interfering with glycolysis, fatty acid oxidation, the tricarboxylic acid cycle (TCA) cycle, and oxidative phosphorylation. BCAAs can directly activate the mammalian target of rapamycin (mTOR) signaling pathway to induce insulin resistance, or function together with fatty acids. In addition, elevated levels of BCAAs and fatty acids can activate the canonical nuclear factor-κB (NF-κB) signaling pathway and inflammasome and regulate mitochondrial dysfunction and metabolic disorders through upregulated inflammatory signals. This review provides a comprehensive summary of the mechanisms through which BCAAs and fatty acids modulate energy metabolism, insulin sensitivity, and inflammation synergistically.

## Introduction

Carbohydrates, lipids, and amino acids are the three major nutrients for humans. They are oxidized, and they supply energy in various ways to maintain activities of the body. When the internal and external environments change, they can interact and transform to each other to adjust various metabolic activities to maintain human health. Once metabolic homeostasis is perturbed, it will cause many endocrine disorders and metabolic diseases, which threaten public health seriously with the increasing morbidity rate and younger trend (1). The pathogeneses of endocrine and metabolic diseases are complex, and the development of multiple disorders are associated with energy metabolism imbalance, chronic low-grade inflammation, and genetic influences (1–4). Recently, alterations in several metabolic pathways have been implicated in the development of metabolic diseases by metabolomic studies (1, 5–9).

Notably, the abnormal levels of branched-chain amino acids (BCAAs) and fatty acids in the circulation are closely related to the progression of metabolic diseases and prognosis, such as obesity, metabolic syndrome, type 2 diabetes mellitus (T2DM), cardiovascular disease, non-alcoholic fatty liver disease, and reproductive endocrine disease (10–19). Insulin resistance (IR) is a common pathophysiological basis of these metabolic diseases (20–24). Mitochondrial dysfunction and inflammation have been considered as key factors of IR (25–27). As important metabolic substrates, BCAAs could induce IR and T2DM by reducing insulin secretion and glucose utilization (28, 29). Moreover, elevated levels of fatty acids could interfere with the insulin signal transduction through regulating inflammation and oxidative stress, leading to the occurrence of IR (30, 31). Thus, both BCAAs and fatty acids play a crucial role in regulating the metabolic homeostasis.

**Figure 1:**
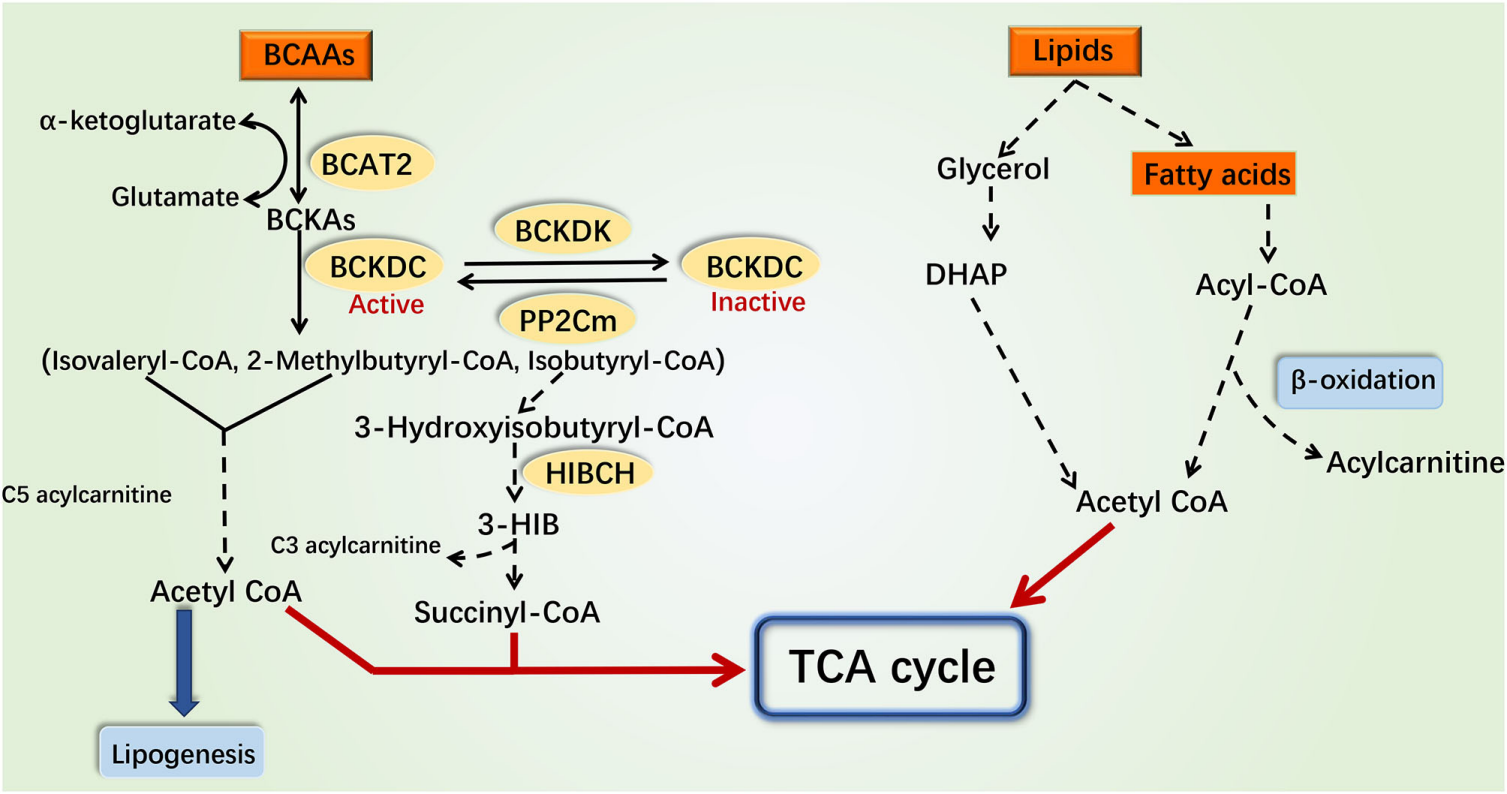
Catabolism pathways of branched-chain amino acids and fatty acids. Various intermediate metabolites produced by the catabolism of BCAAs and fatty acids can participate in the TCA cycle and glycolysis. The catabolism of BCAAs and fatty acids can interplay through these metabolites and ultimately impact the production of mitochondrial ATP through the electronic transport chain. The catabolism of BCAAs in the mitochondria is shown on the left, while the catabolism of fatty acids in cytoplasm and mitochondria is shown on the right. The dotted line indicates a multistep reaction, and the solid line indicates a one-step reaction. DHAP, dihydroxyacetone phosphate; 3-HIB, 3-hydroxyisobuterate; HIBCH, 3-hydroxyisobutyryl-coenzyme A hydrolase.

BCAAs include leucine, isoleucine, and valine. In the mitochondrial matrix, BCAAs first undergo reversible transamination to form the corresponding branched-chain α-keto acids (BCKAs) by branched-chain aminotransferase 2 (BCAT2). The branched-chain α-keto acid dehydrogenase complex (BCKDC) then catalyzes the oxidative decarboxylation of BCKAs to form the corresponding acetyl-CoA derivatives (32, 33), which is the first rate-limiting step in BCAA catabolism. BCKDC is a multienzyme complex, consisting of branched-chain α-keto acid dehydrogenase E1α (BCKDHA), branched-chain α-keto acid dehydrogenase E1β (BCKDHB), branched-chain α-keto acid dehydrogenase E2, and branched-chain α-keto acid dehydrogenase E3 (34). Thereafter, the acetyl-CoA derivatives go through several steps to generate the corresponding metabolites and participate in different metabolic processes (Figure 1). BCAAs and their intermediate metabolites such as C3 and C5 acylcarnitine, acetyl-CoA, succinyl-CoA, and 3-hydroxyisobutyrate (3-HIB), for example, are involved in insulin signal transduction, fatty acid oxidation, tricarboxylic acid (TCA) cycle, glycolysis, mitochondrial biogenesis, inflammation, and other physiological processes (35–37).

Lipids is the general term for fats and lipoids. Neutral fats, also called triglycerides, can be broken down into glycerol and fatty acids in the cytoplasm. Glycerol is converted into dihydroxyacetone phosphate that participates in gluconeogenesis or TCA cycle (38). According to chain length, fatty acids can be classified into long-chain fatty acids, medium-chain fatty acids, and short-chain fatty acids (39). Mitochondrial fatty acid oxidation is an important pathway for maintaining energy homeostasis. Medium- and short-chain fatty acids enter the mitochondria directly for oxidation, while long-chain fatty acids are first activated to long-chain acyl-CoA and then transported into mitochondria by the carnitine shuttle (40, 41). Following dehydrogenation, hydration, dehydrogenation, and thiolysis, the long-chain acyl-CoA derivatives are finally decomposed into acetyl-CoA which participates in the TCA cycle. The metabolic by-products NADH and FADH2 enter the electronic respiratory chain to produce ATP [Figure 1; (17)].

Currently, researches indicated that BCAA catabolism and fatty acid oxidation could interact with each other. Disorders of fatty acid oxidation increased the plasma levels of BCAAs (42). Besides, the accumulation of BCAA intermediate metabolites such as C3, C5 acylcarnitine, and acetyl-CoA inhibited the complete oxidation of fatty acids (33, 37). These findings indicated that the coordinated roles between BCAAs and fatty acids may be critical for the pathogenesis of several metabolic diseases. Therefore, in this review, we focused on the function and molecular mechanism of coordinated modulation of mitochondrial function, IR, and inflammation by BCAAs and fatty acids, in order to better understand the pathogenesis of endocrine and metabolic diseases.

## BCAAs and Fatty Acids Regulate Mitochondrial Function

### Effects of BCAAs and Fatty Acids on Mitochondrial Biogenesis

The mitochondrial biogenesis is mainly regulated in three aspects (43, 44): first, the changes of mitochondria mass or abundance; second, the expression changes of mitochondrial component genes, including cytochrome C (CytC), adenine nucleotide translocase type 1 (ANT1), and β subunit of the mitochondrial H+-ATP synthase (β-F1-ATPase); and third, the transcriptional modulations of genes related to mitochondrial biogenesis. Peroxisome proliferator-activated receptor γ coactivator 1α (PGC-1α) has been extensively described as a master regulator of mitochondrial biogenesis in a variety of tissues (43, 45). Previous studies have shown that activated PGC-1α promotes mitochondrial biogenesis in two ways. The elevated PGC-1α protein level induced an increase in transcription activity of estrogen-related receptor α (*Err*α) and GA repeat-binding protein α (*Gabpa*) on their own promoters, which facilitated the gene expression of downstream nuclear respiratory factor 1 (NRF-1) and upregulated nuclear genes required for mitochondrial biogenesis (46), such as mitochondria transcription factor B1 (TFB1M) and TFB2M. Besides, it promoted the expression of mitochondrial transcription factor A (TFAM) mRNA to increase mtDNA copy number (43, 44).

BCAAs can induce mitochondrial biogenesis in different mechanisms according to different treatment conditions (Supplementary Table 1). In human HepG2 cells, BCAAs can affect mitochondrial biogenesis through protein acetylation and phosphorylation modification and transcriptional regulation. A mixture of BCAAs could enhance the phosphorylation of Ser1177 on endothelial nitric oxide synthase (eNOS), resulting in the activation of eNOS, which led to an increase in nitric oxide (NO) production (47). NO could induce nicotinamide adenosine dinucleotide (NAD)-dependent deacetylase sirtuin1 (SIRT1) expression, which could deacetylate eNOS and PGC-1α, thereby promoting mitochondrial biogenesis (47). In addition, NO activated by BCAAs promoted the PGC-1α expression at the transcription level through cGMP (43, 48). Similarly, leucine can also promote mitochondrial biogenesis through protein post-translational modification and transcriptional regulation. First, in skeletal muscle cells, leucine could enhance the gene expression level of SIRT1, then SIRT1 deacetylated and enhanced liver kinase B1 (LKB1) activity, LKB1 phosphorylated AMP-activated protein kinase (AMPK), and activated AMPK increased the level of NAD+ in cells by upregulating nicotinamide phosphoribosyltransferase (Nampt), thereby activating SIRT1. AMPK and SIRT1 further activated PGC-1α through phosphorylation and deacetylation, respectively (49). Secondly, the regulatory-associated protein of mTOR (raptor) is a defining component of the mTOR complex, and leucine could promote the activation of the mTOR–raptor complex (50). mTOR and raptor interacted with the transcription factor Ying-Yang 1 (YY1), and YY1 was recruited to the promoter region of the gene encoding PGC-1α to induce transcription (46). Collectively, BCAA mixture can activate eNOS and promote the gene expression of PGC-1α through two pathways: (1) the eNOS/NO/SIRT1 pathways and (2) the eNOS-Ser1177/NO/cGMP pathway. Leucine can increase the gene expression of PGC-1α through three pathways: (1) leucine induced PGC-1α expression by activation of the SIRT1/LKB1/AMPK pathway; (2) leucine directly activated SIRT1 to promote PGC-1α expression; and (3) leucine activated mTOR, and mTOR controlled mitochondrial gene expression through the direct modulation of YY1-PGC-1α activity. In addition, both BCAA mixture and leucine could increase the mitochondrial content and induce the expression of TFAM and mitochondrial component genes. However, several studies have shown that valine did not affect the mitochondrial biogenesis (49, 51, 52).

Long-chain, medium-chain, and short-chain fatty acids regulate the expression of PGC-1α through different molecular mechanisms (Supplementary Table 1). PGC-1α interplays with peroxisome proliferators-activated receptor γ (PPARγ) as PPARγ coactivator, and some medium-chain fatty acids (MCFAs) and short-chain fatty acids (SCFAs) could activate PPARγ and promote PGC-1α transcription to modulate mitochondrial biogenesis (53–57). Acetate treatment activated G-protein-coupled receptor 43 (GPR43), and GPR43 further activated ERK1/2 and cAMP response element-binding protein (CREB) in differentiated brown adipocytes, significantly increasing the mRNA expression levels of PGC-1α (57, 58). Supplementation of butyrate elevated PGC-1α expression at mRNA and protein levels by activating the ERK1/2-CREB signaling pathway in brown adipocyte (57, 58). Long-chain fatty acids (LCFAs) had a weaker ability to promote mitochondrial biogenesis through PGC-1α, compared to MCFAs (59). However, different LCFAs have opposite effects on regulating PGC-1α expression (Supplementary Table 1). The n-3 long-chain unsaturated fatty acids, such as eicosapentaenoic acid (EPA) and docosahexaenoic acid (DHA), enhanced the PGC-1α expression and induced mitochondrial biogenesis in adipocytes (60). EPA stimulated phosphorylation and activation of AMPK signaling and upregulated PGC1-α expression in human subcutaneous adipocytes (61). In contrast, some studies have found that certain LCFAs could reduce or not affect the expression of PGC-1α. A mixture of long-chain saturated fatty acids and unsaturated fatty acids stimulated the production of reactive oxygen species (ROS) in adipocyte, which suppressed the expression level of PGC-1α mRNA (62). A long-chain saturated fatty acid palmitate downregulated PGC-1α expression by activating the extracellular signal-regulated kinases 1 and 2 (ERK1/2), mitogen-activated protein kinase (MAPK), and nuclear factor-kappaB (NF-κB) signaling pathways and inhibited mitochondrial biogenesis in skeletal muscle cells (63). The downregulation of PGC-1α mRNA expression might be caused by binding to transcriptional complex I containing the p65 subunit of NF-κB (64). Moreover, the accumulation of fatty acids in obese patients and animal models led to the lowered secretion of adiponectin (61, 65–68). In skeletal muscle cells, adiponectin increased the expression of calcium/calmodulin-dependent protein kinase (CaMK) by activating AMPK, and CaMK induced the expression of PGC-1α (69). The elevated level of adiponectin also inhibited the activity of BCKDC and the expression of PP2Cm, which suppressed BCAA catabolism, increased the BCAAs levels, and further hindered mitochondrial biogenesis (65). Furthermore, in the animal liver tissues, intervention with a high-protein diet (BCAAs significantly increased) plus high-fat diet induced the expression of PGC-1α and TFAM, and in skeletal muscle tissues, leucine supplementation plus high-fat diet also promoted the expression of PGC-1α and TFAM, which suggested the coordinated effect of BCAAs and fatty acids on mitochondrial biogenesis (70, 71).

Overall, BCAAs and fatty acids can regulate the expression of PGC-1α through different mechanisms to influence mitochondrial biogenesis. Differential expression patterns of key enzymes in BCAA catabolism and fatty acid oxidation in different tissues may be the reason that distinguished the regulatory mechanisms of BCAAs and fatty acids. BCAT, the enzyme that initiates BCAA catabolism, can exist as the cytoplasmic BCAT1 and the mitochondrial BCAT2. BCAT2 is expressed ubiquitously in most tissues and especially in skeletal muscle, whereas for the second step of BCAA catabolism, the key enzyme BCKDH is most active in the liver (72). In fatty acid oxidation, fatty acids are needed to be first activated by the acyl-CoA synthetases (ACS), which express in a tissue-specific manner. For example, long-chain acyl-CoA synthetase 1 (ACSL1) is generally distributed in liver cells, cardiomyocytes, and adipocytes (73), while ACSL6 is more common in skeletal muscle (74). Different ACS isoforms channel different fatty acids into specific downstream pathways that may induce different biological effects (75). Besides, different types of long-chain fatty acids have distinct, and sometimes opposing, effects on mitochondrial biogenesis. Based on the current researches, we speculated that the mitochondrial biogenesis might be inhibited by saturated long-chain fatty acids, and the unsaturated long-chain fatty acids might play a positive role in promoting mitochondrial biogenesis. This hypothesis and possible mechanism need to be further studied and confirmed.

### Effects of BCAAs and Fatty Acids on Energy Metabolism

**Figure 2:**
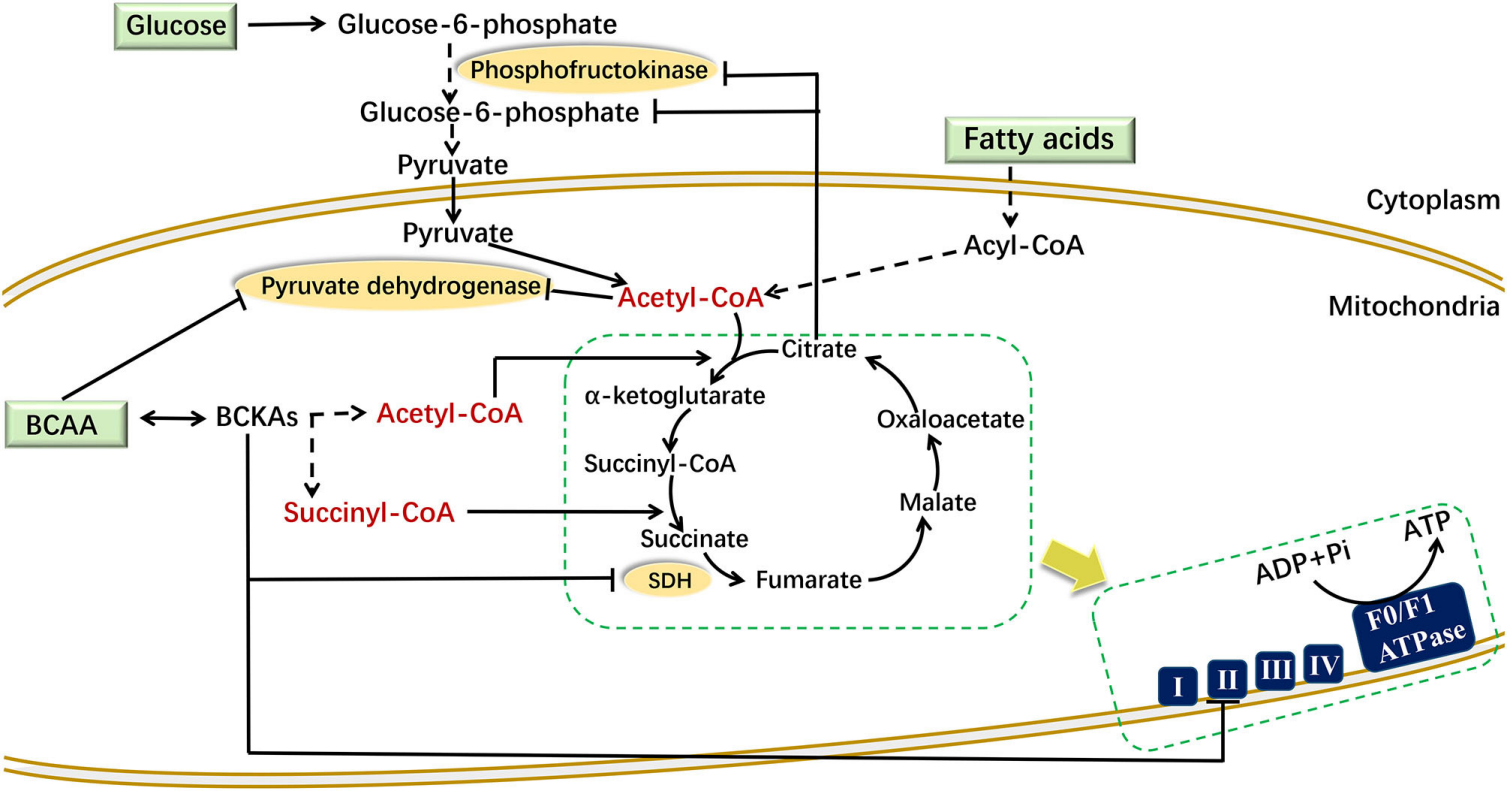
Mechanism of BCAAs and fatty acids regulating energy metabolism. BCAAs and fatty acids affect mitochondrial energy metabolism through different mechanisms in different cells. In mouse heart perfusate, BCAAs inhibited the activity of pyruvate dehydrogenase. In muscle cells, the increased citrate inhibits the accumulation of phosphofructokinase and glucose-6-phosphate. In liver cells, BCKAs can directly suppress the expression of respiratory complex II/SDH to reduce the production of ATP. In the figure, the dotted line indicates a multistep reaction, and the solid line indicates a one-step reaction. SDH, succinate dehydrogenase.

Under aerobic conditions, pyruvate produced by glycolysis enters the TCA cycle to produce carbon dioxide and water. The metabolic by-products NADH and FADH2 undergo oxidative phosphorylation to produce ATP, which maintains the normal energy metabolism of cells. Increased BCAA and fatty acid levels can affect glycolysis (Figure 2). When adding BCAAs to PP2Cm germ-line knockout (KO) mouse heart perfusate, BCAAs inhibited the activity of pyruvate dehydrogenase directly to affect the glycolysis (76). Moreover, enhanced fatty acid oxidation increased the ratio of mitochondrial acetyl-CoA:CoA and NADH:NAD+, leading to the inactivation of pyruvate dehydrogenase. This in turn increased the intracellular level of citrate, thereby inhibiting the accumulation of phosphofructokinase and glucose-6-phosphate, which hindered the normal progress of glycolysis (77).

Increased BCAA and fatty acid levels can also disturb the TCA cycle (Figure 2). The carbon produced by BCAA catabolism and fatty acid oxidation enters the TCA cycle in the form of acetyl-CoA or succinyl-CoA. The succinate dehydrogenase catalyzes the conversion from succinate to fumarate in the TCA cycle, and it is also the respiration Complex II in the electron transfer chain. In liver cells, the elevated BCKA levels suppressed the gene expression of succinate dehydrogenase to block the TCA cycle and ATP production (78). In high-fat-diet (HFD) mice, enhanced fatty acid oxidation increased the amount of acetyl-CoA entering the TCA cycle in the early period. While due to the “overload” of the TCA cycle, the flux of acetyl-CoA decreased in the following stage (79).

Increased levels of BCAAs and fatty acids may affect mitochondrial oxidative phosphorylation (OXPHOS) through their common target, PGC-1α. PGC-1α enhanced the expression of *Err*α and *Gabpa* and then activated the expression of downstream OXPHOS-related genes (80). These genes mainly included two categories: (1) mitochondrial OXPHOS-related enzyme-encoding genes, such as cytochrome c oxidase 1-5 (COX 1-5), ATP synthase (ATP5G), citrate synthase (CS), ATP synthase peripheral stalk subunit OSCP (Atp5o), succinic dehydrogenase (SDH), and malate dehydrogenase (MDH), and (2) mitochondrial substrate oxidation-related regulators, such as PPARβ/δ and cyclin-dependent kinase 4 (CDK4). In addition, NRF-1 activated by PGC-1α could combine with the promoters of the OXPHOS-related genes to stimulate their transcription levels (45, 62). Therefore, BCAAs and several fatty acids could synergistically enhance mitochondrial OXPHOS through upregulating PGC-1α expression. However, elevated BCAAs and fatty acids can also inhibit ATP production through other mechanisms. In mouse C2C12 myoblast cells, although leucine promoted the expression of PGC-1α and increased OXPHOS, a decrease in glycolysis and ATP content was observed (81). In PP2Cm knockout mouse liver, increased BCKA levels inhibited the expression of respiratory complex II, which subsequently interfered with both mitochondrial OXPHOS and ATP production [Figure 2; (78)]. In addition, a study suggested that treatment of skeletal muscle cells with a low concentration of 0.5–2 μM free fatty acids stimulated ATP production (82), while treatment of adipocytes with a high concentration of free fatty acids >5 μM inhibits ATP production (62). We speculate that a potential reason may be that the condition with low concentration of fatty acids is closer to the physiological state, while the condition with high levels of fatty acids is in the pathological state, therefore having different pathophysiology effects. The mechanism by which different pathophysiology effects of fatty acids on OXPHOS and ATP production requires further research.

## BCAAs and Fatty Acids Regulate Inflammatory Signals

### BCAAs and Fatty Acids Upregulate Inflammation, and BCAA Catabolism Is Affected by Inflammatory Signals

**Figure 3:**
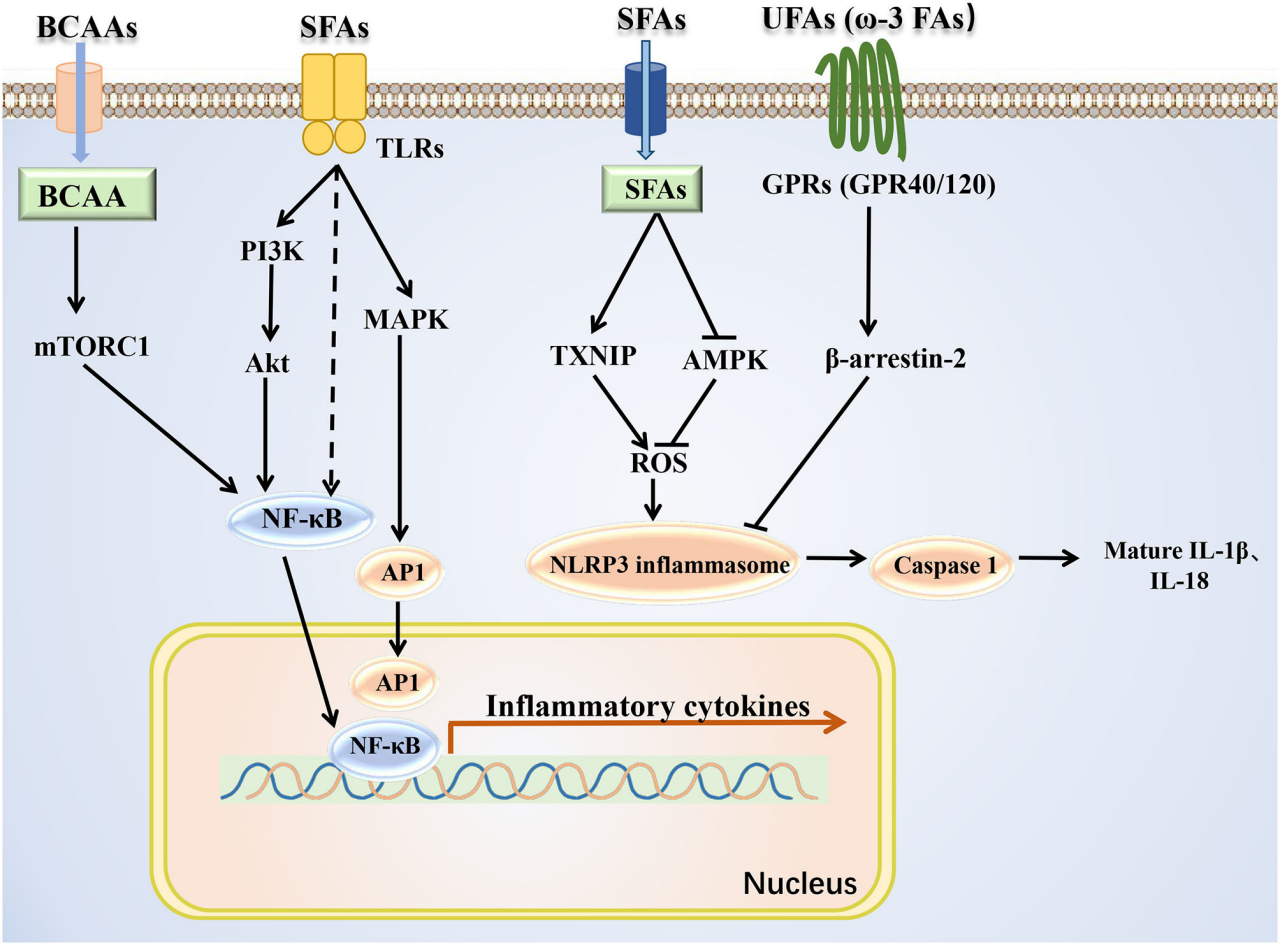
Mechanism of BCAAs and fatty acids regulating inflammatory signals. BCAAs and different types of fatty acids regulate the inflammatory response through the NF-κB pathway and NLRP3. SFAs, saturated fatty acids; UFAs, unsaturated fatty acids; TXNIP, thioredoxin-interacting protein.

BCAAs and fatty acids can induce the inflammation (Figure 3). Supplementation with BCAAs could activate mTORC1 and upregulate the NF-κB signaling pathway, increasing the release of pro-inflammatory cytokines in human peripheral blood mononuclear cells and endothelial cells (83, 84). Treatment with the saturated fatty acid palmitate in C2C12 cells rapidly induced the association of myeloid differentiation factor 88 (MyD88) with the toll-like receptor 2 (TLR2), increased the degradation of IkappaB and NF-κB DNA binding, and enhanced interleukin (IL)-6 production (85). In addition, saturated (lauric acid) and polyunsaturated (DHA) fatty acids reciprocally modulated the activation of TLR4 and its downstream signaling pathways involving PI3K/Akt/NF-κB in macrophages (86), further suggesting the possibility that TLR-mediated target gene expression and cellular inflammatory responses are also differentially mediated by saturated and unsaturated fatty acids. Besides, studies have shown that several types of fatty acids can activate the inflammasome. Saturated fatty acid palmitate, but not unsaturated oleate, activated ROS through inhibiting AMPK, induced the activation of the Nod-like receptor pyrin domain containing 3 (NLRP3) inflammasome, and caused IL-1β and IL-18 production in hematopoietic cells (87). Saturated fatty acids could also directly activate the NLRP3 inflammasome through upregulating thioredoxin-interacting protein (TXNIP) in HFD-induced mice (88). By contrast, omega-3 polyunsaturated fatty acids (ω-3 FAs), including EPA and DHA, alleviated the inflammation by preventing NLPR3 activation in an HFD-induced model. The G protein-coupled receptors (GPR120 and GPR40) and the downstream protein β-arrestin-2 (ARRB2) were shown to be involved in inflammasome inhibition induced by ω-3 FAs (89). Overall, saturated and unsaturated fatty acids may play pro-inflammatory and anti-inflammatory roles, respectively, at the level of transcriptional regulation and protein processing of inflammatory factors.

### BCAAs and Fatty Acids Regulate Mitochondrial Dysfunction Through Inflammatory Signals

Previous studies have indicated that inflammatory signals could influence the mitochondrial biogenesis by decreasing PGC-1α expression. In human cardiac cells, tumor necrosis factor-α (TNF-α) reduced PGC-1α expression mediated via both p38 MAPK and NF-κB pathways, and PGC-1α downregulation resulted in a reduction in pyruvate dehydrogenase kinase 4 (PDK4) expression and an increase in glucose oxidation rate (64). The excessive TNF-α bound to the p55 subtype of TNF receptor, and then inhibited eNOS activation and reduced NO level in obese rodents, leading to a decrease in the expression of PGC-1α (90). In addition, treatment 3T3-L1 adipocytes with TNF-α induced the downregulation of the mRNA expression of many TCA circulation-related enzyme genes, such as *Aco2, Idh2, ogdh*, and *Fh1* (91). Thus, the coordinated activation of inflammatory signals by BCAAs and some fatty acids may interfere with markers of mitochondrial biogenesis mitochondrial biogenesis and energy metabolism, even leading to metabolic disorders.

## BCAAs and Fatty Acids Regulate Insulin Sensitivity

### BCAAs and Fatty Acids Affect Insulin Signal Transduction

**Figure 4:**
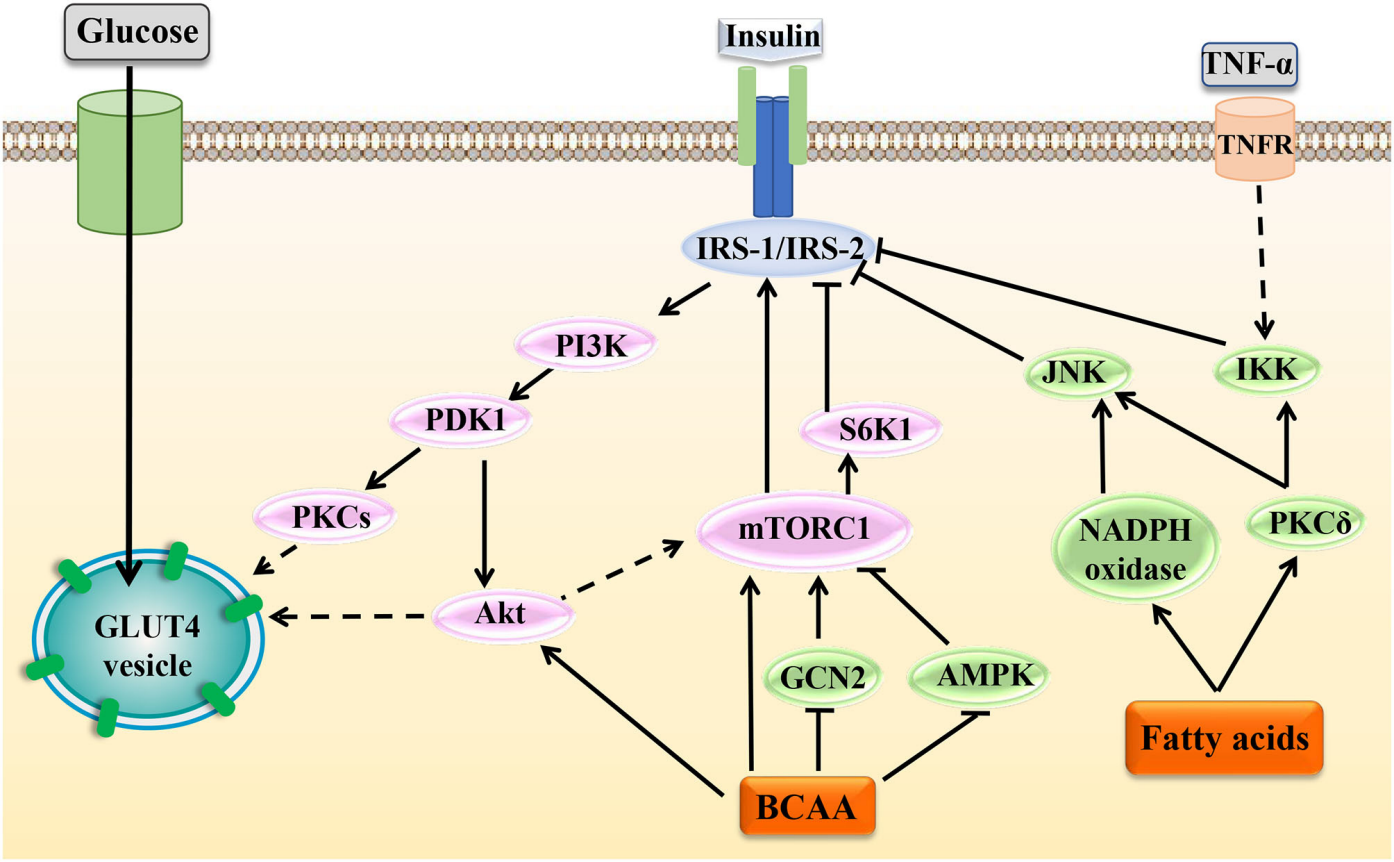
Effects of BCAAs and fatty acids on insulin signal transduction. Under normal circumstances, insulin can activate various molecules such as PI3K, Akt, and mTOR to affect the activation of IRS and regulate the transport and ectopic expression of GLUTs. Increased levels of BCAAs and fatty acids can interfere with normal insulin signaling through various mechanisms and ultimately lead to IR. In the figure, the dotted line indicates a multistep reaction, and the solid line indicates a one-step reaction.

Insulin is the key hormone controlling glucose and lipid metabolism. Under normal physiological conditions, secreted insulin binds to the insulin receptor, fostering tyrosine phosphorylation of the insulin receptor substrate (IRS)-1/2 and regulating downstream cascade signals. Several kinases have been implicated in this process, including PI3K, 3-phosphoinositide-dependent protein kinase (PDK), Akt, and S6K1. Feedback control in insulin signaling involves serine phosphorylation of IRS proteins, which inhibits tyrosine phosphorylation and blocks the ectopic expression and translocation of glucose transporters (GLUTs) and ultimately produces IR [Figure 4; (92)]. Some studies have shown that BCAAs, especially leucine, promoted insulin signal transduction. Leucine induced the tyrosine phosphorylation of IRS-1 and improved insulin-stimulated Akt and mTOR phosphorylation, preventing HFD-induced IR in insulin-target tissues (93). The possible mechanism is that BCAAs or leucine-induced protein synthesis were accompanied by energy expenditure, leading to an increase in insulin signal transduction, GLUT4 content, and glucose uptake (94–96). However, some studies have shown that leucine could inhibit insulin signal transduction through other mechanisms (Figure 4). Incubation of rat skeletal muscle with elevated concentrations of leucine suppressed AMPK activation and concomitantly increased mTOR/p70S6K signaling and led to IR (97). Additionally, leucine deprivation could improve hepatic insulin sensitivity by sequentially activating general control non-derepressible (GCN) 2, an amino acid sensor, and decreasing mTOR/ p70S6K signaling (98). The specific mechanisms of BCAAs in regulating the insulin sensitivity and glucose metabolism in different conditions need further exploration.

Fatty acids can influence IRS activity through regulating protein phosphorylation of IRS or transcriptional level of IRS via histone acetylation. In adipocytes, fatty acids activated PKCδ, leading to activation of serine kinase inhibitor kappaB kinase (IKK) and c-JUN NH2 terminal kinase (JNK), which catalyzed the phosphorylation of IRS-1, ultimately reducing the insulin-induced glucose uptake (99). Fatty acids induced oxidative stress by activating PKCδ and NADPH oxidase, increased JNK phosphorylation, and thereby enhanced serine phosphorylation of IRS-1 and IRS-2 and impaired hepatic insulin signal transduction (100). However, sodium butyrate, the sodium salt form of butyric acid, is hydrolyzed to form a shortchain fatty acid butyric acid. Butyric acid could act as the histone deacetylase inhibitor and favor insulin sensitivity, and butyrate improved palmitate-induced IR by increasing histone H3 acetylation on chromatin in proximity of the *Irs1* transcriptional start site in L6 myotubes, indicating that a certain SLFA-mediated insulin-sensitizing action was dependent on epigenetic effects (101).

### BCAAs and Fatty Acids Induce IR Indirectly by Affecting Mitochondrial Function

PGC-1α- and PGC1α-responsive genes involved in OXPHOS were downregulated in skeletal muscles of patients with IR (45, 80), which suggested that reduced mitochondrial biogenesis and energy metabolism were closely related to IR. Moreover, PGC-1α could induce valine catabolism to produce intermediate 3-Hydroxyisobutyrate (3-HIB), and 3-HIB acted as a paracrine factor to reduce insulin sensitivity by inhibiting Akt phosphorylation in C2C12 myotubes (102). Besides, plasma concentrations of BCAAs and 3-HIB were inversely related to insulin sensitivity in overweight to obese individuals, while changes in 3-HIB rather than changes in BCAAs were associated with metabolic improvements with weight loss (103), supporting a crucial role of 3-HIB in the development of insulin resistance (104, 105). In addition, elevated levels of acylcarnitine, a product of incomplete oxidation of BCAAs and fatty acids, caused mitochondrial stress, which interfered with insulin signal transduction (7, 106, 107). However, recent findings indicated that abnormal mitochondrial function was not an early event in the development of IR but an adaptive response to excess nutrients in the body (71). Further research is needed to clarify the regulatory mechanisms of BCAAs and fatty acids in affecting insulin sensitivity.

### BCAAs and Fatty Acids Induce IR Indirectly by Affecting Inflammation Signals

An increasing body of evidence has shown that chronic low-grade inflammation participated in the development of IR [Figure 4; (108, 109)]. In skeletal muscle cells, TNF-α activated MAPK, leading to downstream phosphorylation activation of IKK. Then, IKK phosphorylated IRS to cause IR (110). In brown adipocytes, TNF-α could activate MAPK and ERK, which led to the serine phosphorylation of IRS-2 and then caused IR (111). Moreover, in adipocytes, IL-1β inhibited the activation of IRS by phosphorylating JNK or MAPK. Besides, IL-1β could partially inhibit the activation of IRS-1 by activating ERK (112). In adipocytes, IL-6 reduced the protein expression of the insulin receptor β subunit and IRS-1 and simultaneously downregulated the expression of GLUT4. Besides, IL-6 could suppress the insulin-stimulated Akt/PKB and ERK1/2 activation (113). Also, research results showed that in skeletal muscle cells, fatty acids activated the MAPK, JNK, and NF-κB pathways by binding to TLR2, which inhibited IRS tyrosine and Akt phosphorylation, finally inducing IR. At the same time, the activation of this pathway induced the production of the inflammatory cytokine IL-6, which further aggravated the occurrence and development of IR (85). Therefore, as mentioned above, BCAAs and some fatty acids could activate inflammatory signals and increase the release of inflammatory cytokines; thus, they may exacerbate the development of IR by blocking insulin signaling transduction in adipocytes and skeletal muscle cells.

## Increased BCAAs and Fatty Acids Levels Are Closely Related to the Occurrence of Various Metabolic Diseases

In clinical studies, there was a positive correlation between homeostatic model assessment (HOMA) index, glycated hemoglobin (HbA_1c_), and increased BCAAs levels in the plasma (106). Furthermore, increased IR and proteolysis could result in elevated plasma levels of BCAAs in patients with NAFLD (114). In addition, there was a significant reduction of the BCAA catabolic enzymes BCKDHA, BCKDHB, and BCAT2 in the visceral white adipose tissue of obese people with metabolic syndrome, leading to an increase in BCAA levels in the circulation (115), while the expression of BCAA catabolic enzymes in the adipose tissue was negatively correlated with IR (116).

Excess lipids inhibited the complete oxidation of fatty acids in the mitochondria (107). Different types of fatty acids could regulate insulin sensitivity through distinct mechanisms (17). In patients with metabolic syndrome, enhanced lipolysis of the adipose tissue led to elevated levels of fatty acids in the circulation, disturbing the insulin signals and generating the phenotypes of IR and obesity (117). Ectopic fatty acid accumulation was an early manifestation of NAFLD. As the disease progresses, oxidation of free fatty acid was decreased in the liver, producing toxic metabolites such as diacylglycerol and ceramide (118). Chronic low-grade inflammation was one of the main causes of IR in T2D and obese patients (119, 120). BCAAs and fatty acids could also mediate the occurrence of IR by activating inflammatory cytokines and inflammatory signaling pathways (83, 84, 121).

Additionally, metabolic disorders of BCAAs and fatty acids could also affect the reproductive function. Polycystic ovary syndrome (PCOS) is one of the most common reproductive endocrine diseases and the leading cause of anovulatory infertility. PCOS patients are accompanied by obvious metabolic abnormalities and chronic inflammation and have a higher risk for diabetes and cardiovascular disease compared with the healthy women (16). Previous studies have shown that the levels of BCAAs and fatty acids in both plasma and follicular fluids were significantly increased in PCOS patients (15, 16, 102, 103, 122). Moreover, the increased BCAA levels in the follicular fluid was negatively associated with the pregnancy outcome (103), which suggested that the systemic metabolic disorders of BCAAs could alter metabolic homeostasis of the follicular microenvironment for oocyte maturation and embryo development. Furthermore, there were mitochondrial dysfunction and inflammation in the ovarian granulosa cells of PCOS patients, affecting the microenvironment of follicular development (123–129), but the regulatory mechanism had not been elucidated clearly. Therefore, the modulation of mitochondrial function and inflammation by BCAAs and fatty acids may be helpful for us to comprehensively explore the pathogenesis of PCOS, so as to provide new ideas and targets for clinical diagnosis and treatment.

## Conclusion

In summary, elevated levels of BCAAs and fatty acids can regulate cell metabolism by affecting mitochondrial function and inflammation signals. Mitochondrial dysfunction, inflammation, and IR can further lead to the accumulation of BCAAs and fatty acids, thus aggravating the development of metabolic diseases. However, there are still many issues that need further exploration; whether BCAAs and fatty acids synergistically regulate energy metabolism and inflammation through other signaling pathways, whether the mediated mechanisms of BCAAs and fatty acids reported are tissue specific, and whether abnormal levels of BCAAs and fatty acids could be a useful marker for risk prediction and a new target for clinical diagnosis and treatment of metabolic disease. These issues all require further study.

## Author Contributions

ZY collected the information, designed the pictures, and wrote and submitted the manuscript. SW and CZ collected the information and joined in the critical discussion. YZ critically revised the manuscript and contributed to the conception of the design. All authors contributed to the article and approved the submitted version.

## Conflict of Interest

The authors declare that the research was conducted in the absence of any commercial or financial relationships that could be construed as a potential conflict of interest.
