# Supplementary material for: Coordinated Modulation of Energy Metabolism and Inflammation by Branched-Chain Amino Acids and Fatty Acids
Source: Front Endocrinol (Lausanne). 2020 Sep 8;11:617. doi: 10.3389/fendo.2020.00617 (PMC7506139; doi:10.3389/fendo.2020.00617)
Supplement: Supplementary file 1 [file Data_Sheet_1.ZIP › Supplementary Table 1.docx]

**Supplementary Table 1. Effects of different BCAAs types and concentrations on mitochondrial biogenesis and energy metabolism related genes and indicators in different cells.**

Classification 1 indicates changes in quality or abundance of mitochondria; Classification 2 shows changes in genes related to mitochondrial biogenesis; Classification 3 indicates changes in mitochondrial component genes;

Classification 4 represents changes in mitochondrial oxidative phosphorylation genes; Classification 5 shows other related changes.

| **Experimental subject** | **Treatment** | | **Main effects** |
| --- | --- | --- | --- |
| cardiomyocytes | BCAA-enriched mixture | | 1) ↑ mtDNA; TFAM mRNA expression (35).  2) ↑ PGC-1α, NRF-1, SIRT1 mRNA expression, eNOS expression, p-mTOR (35).  3) ↑ CytC, β-F1-ATPase mRNA expression (35).  4) ↑ CS, COX4 mRNA expression, ATP amount (35). |
| Human HepG2 cells | BCAA-enriched mixture | | 1) No effect (47).  2) ↑ PGC-1α, SIRT1 expression; eNOS expression (47).  3) ↑ CytC expression (47).  4) ↑COX4 expression (47). |
| skeletal muscle cell | 0.1mM leucine | | 1) ↑ mitochondrial content (81).  2) ↑ PGC-1α expression (81).  3) ↑ CytC expression (81).  4) ↑ oxidative metabolism, mitochondrial uncoupling (81).  5) ↓ glycolytic metabolism, ATP content (81). |
|  | 0.5mM leucine | | 1) ↑ mitochondria content and mass (49, 81, 136, 137); TFAM mRNA expression (137).  2) ↑ PGC-1α, NRF-1, SIRT1mRNA expression (49, 81, 136, 137); SIRT1 activity, p-AMPK (49).  3) ↑ ANT1 (138); Hspd1 mRNA expression (49); CytC expression (81).  4) ↑ PPARγ (138), COX5b, COX2 (49) mRNA expression; PPARβ/δ, CDK4 expression (137); oxidative metabolism, mitochondrial uncoupling (81).  5) ↑ glycolytic metabolism (81). |
|  | 1.0 mM leucine | | 2) ↑ PGC-1α, SITR3 mRNA expression (139). |
|  | 1.5mM leucine | | 1) ↑ TFAM mRNA expression (138).  2) ↑ PGC-1α, NRF-1, SIRT1 mRNA expression; p-mTORC1 (138).  4) ↑ PPARγ mRNA expression (138). |
|  | 2 mM L-leucine/leucine | | 1) ↑ mitochondrial content, TFAM expression (51, 52, 139, 140); TFB1M mRNA expression (52).  2) ↑ PGC-1α, NRF1, SIRT1 expression (51, 52, 139, 140); p-AMPK, p-LKB1 (52).  3) ↑ CytC expression (51, 52).  4) ↑ PPARβ/δ (51); CS (140); COX I, ATP5G expression, SDH, MDH activity (52). |
|  | 10 mmol/L L-leucine | | 1) ↑ TFAM mRNA expression (141).  2) ↑ PGC-1α mRNA expression (141).  3) ↑ CytC mRNA expression (141).  4) ↑ Atp5o mRNA expression (141). |
|  | 0.5 mM or 2 mM or 10 mmol/L valine | | No effect (49, 51, 141). |
| 3T3-L1 preadipocytes | 0.5mM leucine | | 1) ↑ mitochondria mass (136).  2) ↑ PGC-1α, SIRT-1, NRF-1 mRNA expression (136).  4) ↑ COX mRNA expression (136). |
| 3T3-L1 preadipocytes | long-chain fatty acid | 1mM long-chain fatty acid mixture | 1) ↓ TFAM mRNA expression (61).  2) ↓ PGC-1α, NRF-1 expression, ROS, SIRT1 protein expression (61). |
| skeletal muscle cell |  | 500μM palmitate | 2) ↓ PGC-1α mRNA expression, ↑ p38 MAPK (142).  3) ↓ ANT1 mRNA expression (142).  4) ↓ CS, Ndufa5, ATP5J2, IDH2 mRNA expression (142). |
|  |  | 0.75 mmol/l palmitate | 2) ↓ PGC-1α expression, ↑ p-MEK1/2, p-ERK, p-65, IκBα (62). |
| adipocytes |  | 100–200 μM eicosapentanoic acid | 1) ↑ mitochondrial content, TFAM mRNA expression (143).  2) ↑ PGC-1α deacetylation, NRF-1, SIRT1 mRNA expression, SIRT1 activity, p-AMPK (143).  4) ↑ COX IV mRNA expression (143). |
|  |  | 6% EPA and 51 % DHA | 2) ↑ PGC-1α, NRF-1 mRNA expression (144).  3) ↑ COX6, ATP5A1 mRNA expression (144).  4) ↑ COX IV mRNA expression (144). |
| brown adipocytes | short-chain fatty acid | acetate | 1) ↑ mtDNA (57).  2) ↑ PGC-1α, PPARγ expression, p-ERK1/2, p-CREB (57). |
| skeletal muscle cell |  | Sodium butyrate | 2) ↑ PGC-1α expression, p-AMPK, p38 (58). |
