# Supplementary figures and images for: Coordinated Modulation of Energy Metabolism and Inflammation by Branched-Chain Amino Acids and Fatty Acids
Source: Front Endocrinol (Lausanne). 2020 Sep 8;11:617. doi: 10.3389/fendo.2020.00617 (PMC7506139; doi:10.3389/fendo.2020.00617)

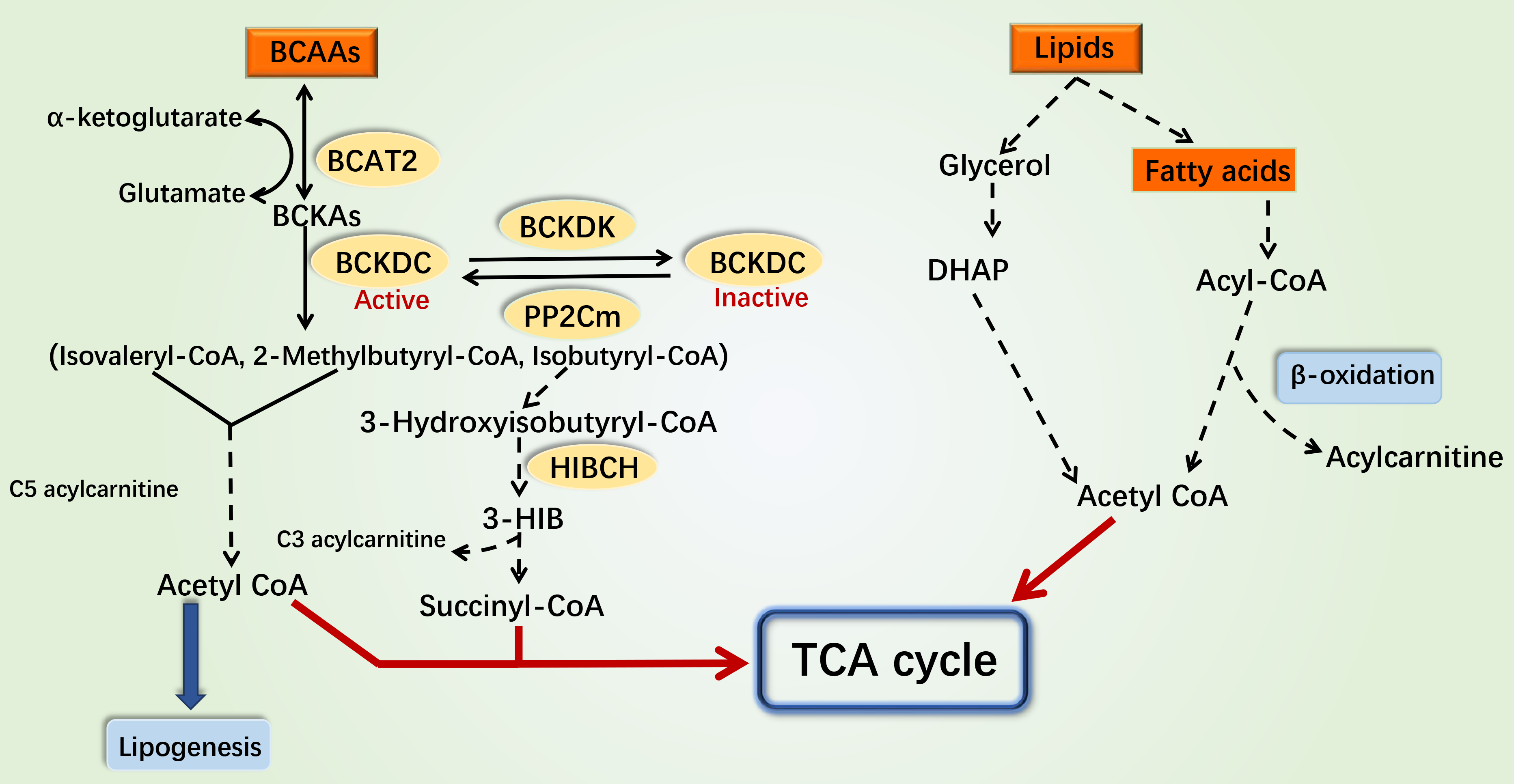

Supplement: Supplementary file 1 [file Data_Sheet_1.ZIP › Revised-figure 1.tif]

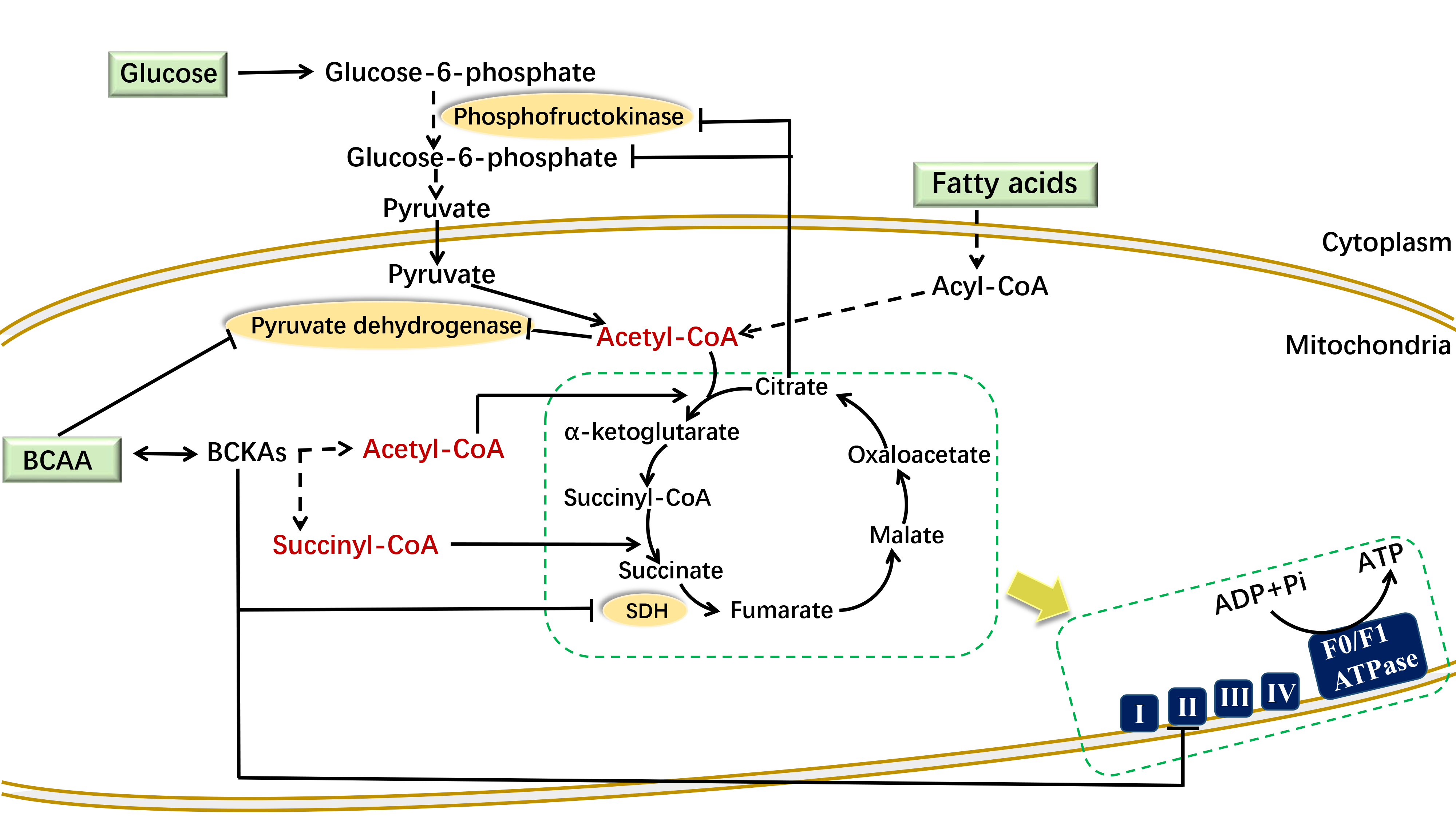

Supplement: Supplementary file 1 [file Data_Sheet_1.ZIP › Revised-figure 2.tif]

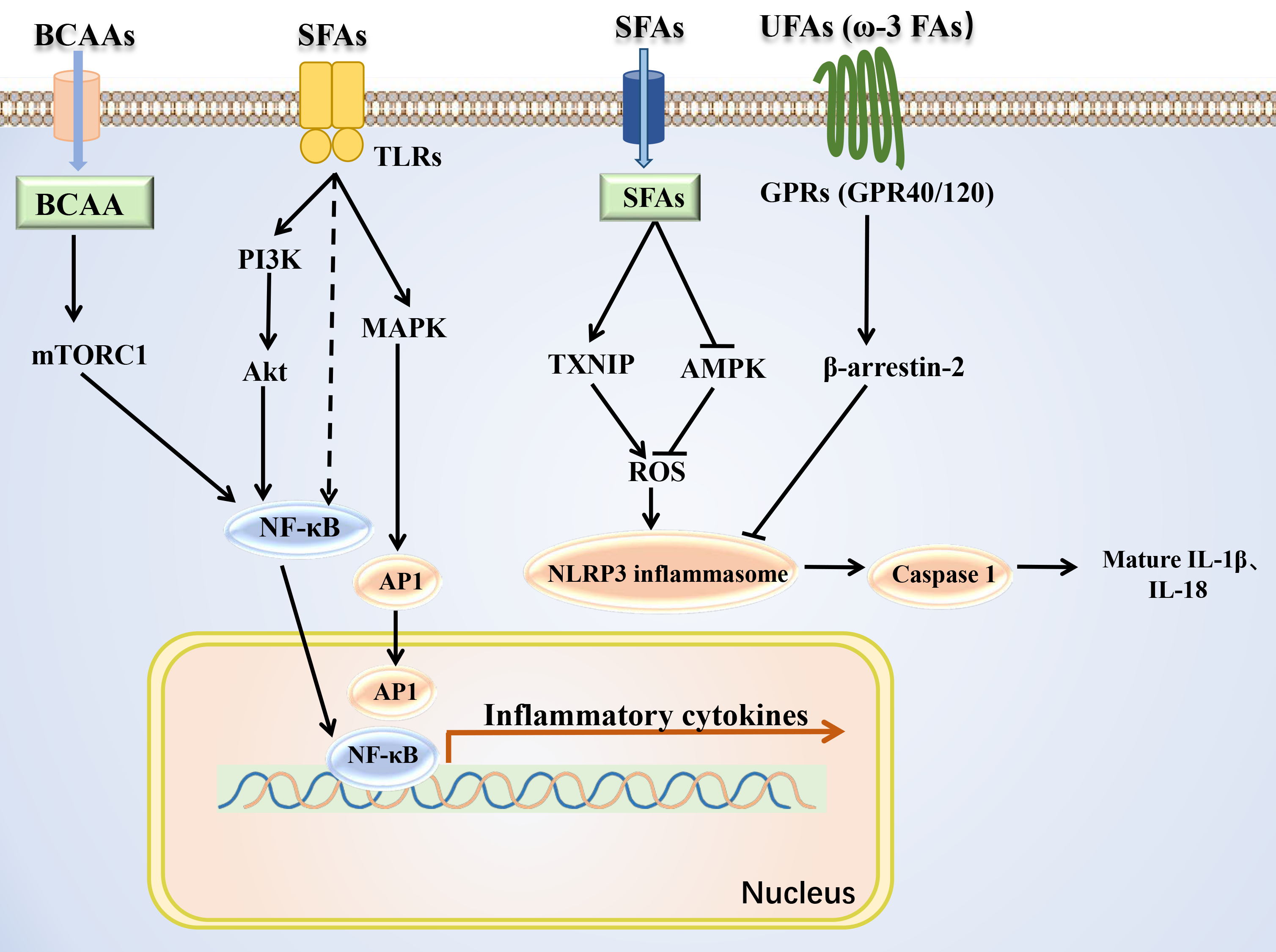

Supplement: Supplementary file 1 [file Data_Sheet_1.ZIP › Revised-figure 3.tif]

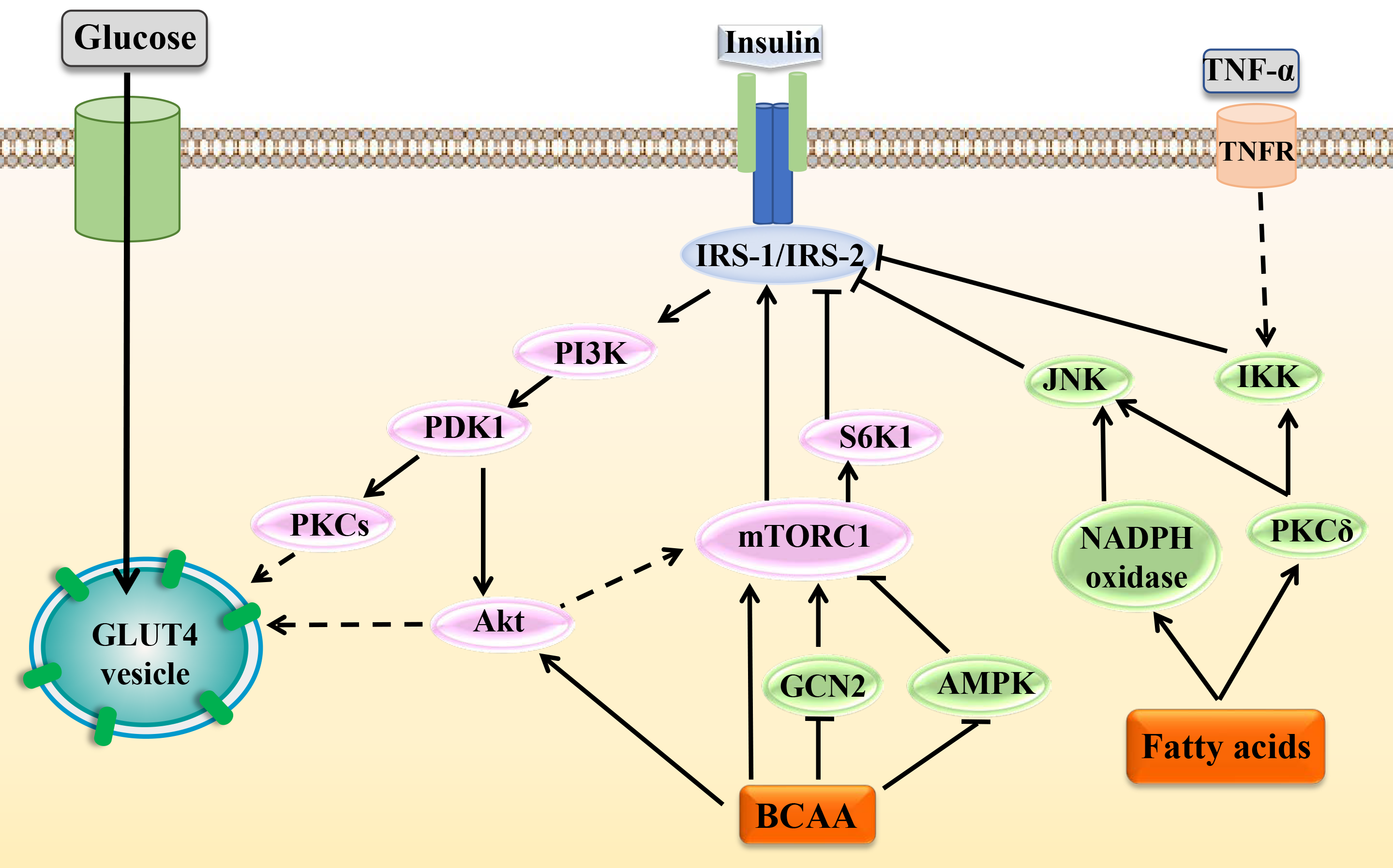

Supplement: Supplementary file 1 [file Data_Sheet_1.ZIP › Revised-figure 4.tif]
